# Efficacy of Neoadjuvant Single or Dual Anti-HER-2 Therapy Combined with Chemotherapy in Patients with HER-2-Positive Breast Cancer: A Single-Center Retrospective Study

**DOI:** 10.31557/APJCP.2021.22.5.1467

**Published:** 2021-05

**Authors:** Qian He, Jia-Yi Li, Qing-Lan Ren

**Affiliations:** *Department of Oncology, The First Affiliated Hospital of Chongqing Medical University, Chongqing, China. *

**Keywords:** Breast cancer, HER-2-positive, anti-HER-2 targeted therapy, pathologic complete response

## Abstract

**Background:**

Studies have shown that neoadjuvant anti-HER-2 therapy and chemotherapy can increase pathologic complete response (pCR) rate in HER-2-positive breast cancer patients and improve prognosis. However, data from Chinese patients are limited. Therefore, we conducted a single-center retrospective study to evaluate the effects of neoadjuvant single or dual anti-HER-2 therapy and chemotherapy in Chinese HER-2-positive breast cancer patients and to explore the prognostic indicators of pCR and progression-free survival (PFS).

**Methods:**

We included patients with HER-2-positive breast cancer treated with neoadjuvant anti-HER-2 therapy and chemotherapy at the First Affiliated Hospital of Chongqing Medical University in China from January 2016 to July 2020. We analyzed the relationship between patient characteristics and the pCR rate or PFS.

**Results:**

Forty-seven patients with HER-2-positive breast cancer receiving neoadjuvant anti-HER-2 therapy and chemotherapy were included. Univariate analysis suggested that compared with patients receiving neoadjuvant single anti-HER-2 therapy, patients receiving neoadjuvant dual anti-HER-2 therapy tended to have a higher pCR rate and better PFS. Patients who achieved pCR also tended to have longer PFS. Multivariate analysis indicated that patients with greater systemic inflammation response index (SIRI) reduction (>0.54) during neoadjuvant treatment (NAT) and patients with a lower T stage were more likely to achieve pCR. Patients aged ≤60 years with lower Ki-67 had longer PFS.

**Conclusion:**

Greater SIRI reduction during NAT was an independent influencing factor for pCR. Patients receiving neoadjuvant dual anti-HER-2 therapy and chemotherapy tended to have higher pCR rates and longer PFS. Patients who achieved pCR also tended to have longer PFS.

## Introduction

Breast cancer is the most common cancer in women and is one of the leading causes of cancer-related death in women (Siegel et al., 2019). With the development and application of anti-HER-2 drugs, the prognosis of breast cancer patients with HER-2 positivity has been significantly improved (Perez et al., 2011). Studies have found that the addition of targeted therapy to preoperative neoadjuvant therapy can improve the prognosis of HER-2-positive breast cancer patients without significantly increasing cardiac toxicity (Petrelli et al., 2011; Schneeweiss et al., 2013). The NeoSphere study found that in patients with HER-2-positive breast cancer, preoperative neoadjuvant chemotherapy (NAC) combined with dual anti-HER-2 therapy (trastuzumab and pertuzumab) significantly increased pathologic complete response (pCR) compared with chemotherapy combined with single anti-HER-2 therapy. Among patients who received preoperative anti-HER-2 therapy, those who achieved pCR had longer PFS than patients who did not achieve pCR (HR=0.54, 95% CI: 0.29-1.00) (Gianni et al., 2016), and the cardiotoxicity of neoadjuvant dual anti-HER-2 therapy combined with chemotherapy was tolerable (Schneeweiss et al., 2013).

In addition to pCR, tumor staging, molecular subtype, histological characteristics, basic patient information, and treatment methods also have an impact on the prognosis of patients with HER-2-positive breast cancer (Ryu et al., 2017; Hwang et al., 2019). In addition, nutritional status can also influence the prognosis of cancer patients (Demark-Wahnefried et al., 2015). The prognostic nutritional index (PNI) is one of the most common parameters for assessing nutritional status. The PNI is equal to 10×serum albumin concentration (g/dL) + 0.005×total lymphocyte count (/mm3)(Mohri et al., 2016). Data have demonstrated that low PNI status before surgery or PNI reduction during NAC is a predictor of poor prognosis in many malignancies, including breast cancer (Tokunaga et al., 2015; Mohri et al., 2016; He et al., 2017; Migita et al., 2017; Hua et al., 2019; Oba et al., 2020). However, the role of PNI in breast cancer patients who received neoadjuvant anti-HER-2 therapy remains unclear. Some studies have also shown a link between cancer-related inflammation and treatment effects (Diakos et al., 2014). The systemic inflammation response index (SIRI: neutrophil count×monocyte count/lymphocyte count) is based on neutrophils, monocytes and lymphocytes and can comprehensively evaluate the balance between immune and inflammatory states in patients. Evidence has shown that the SIRI can predict survival in some patients with malignant tumors (Qi et al., 2016; Li et al., 2017; Hua et al., 2020; Sun et al., 2020; Wang et al., 2020b). However, the prognostic value of the SIRI in neoadjuvant anti-HER-2 targeted therapy for breast cancer remains unclear.

However, much data exists on neoadjuvant anti-HER-2 therapy and chemotherapy in patients with HER-2-positive breast cancer in some countries (Hurvitz et al., 2018; Fasching et al., 2019). However, due to issues such as drug availability and medical insurance coverage, neoadjuvant anti-HER-2 therapy is still not widely used among Chinese patients with HER-2-positive breast cancer (Li et al., 2018), especially neoadjuvant dual anti-HER-2 therapy. Therefore, this study aimed to explore the pCR rate of HER-2-positive breast cancer in Chinese patients receiving neoadjuvant anti-HER-2 therapy (including chemotherapy and trastuzumab and/or pertuzumab) and whether factors including PNI, SIRI, etc., could predict the pCR rate and postoperative PFS in patients with HER-2-positive breast cancer after receiving neoadjuvant anti-HER-2 therapy and chemotherapy.

## Materials and Methods

*Patients and study design*

The search criteria included patients with HER-2-positive primary breast cancer using neoadjuvant anti-HER-2 therapy and chemotherapy at the First Affiliated Hospital of Chongqing Medical University in China from January 2016 to July 2020. The inclusion criteria were as follows: (1) females; (2) diagnosed with HER-2-positive invasive breast cancer (Wolff et al., 2014); (3) neoadjuvant therapy containing chemotherapy and trastuzumab and/or pertuzumab; and (4) patients undergoing surgery after neoadjuvant treatment (5) whether or not to receive radiation and chemotherapy is determined according to the NCCN guidelines. The exclusion criteria were as follows: (1) distant metastasis at initial diagnosis; (2) patients who did not receive neoadjuvant treatment; (3) patients who did not receive surgery; and (4) no regular follow-up. Baseline characteristics and treatments were collected, including age, breast cancer staging, pathology, laboratory tests, imaging tests, treatment, surgery, survival and other data. The primary endpoint was PCR, and the secondary endpoint was PFS.

Neoadjuvant chemotherapy regimens included taxane-based regimens or anthracene-based regimens. Neoadjuvant anti-HER-2 therapy included trastuzumab (8 mg/kg loading dose, then 6 mg/kg, once every 3 weeks) and pertuzumab (840 mg/m^2^ loading dose, then 420 mg/kg, once every 3 weeks). All patients underwent surgery. After surgery, all patients received anti-HER-2 treatment (except for a few patients who stopped targeting due to factors such as disease progression or the COVID-19 epidemic, a total of 1 year of anti-HER-2 treatment was received). The radiation oncologist decided whether to use adjuvant radiation. All hormone receptor-positive patients received adjuvant endocrine therapy. The patients were followed every 3 months.

PCR was defined as no residual invasive cancer cells in the breast or axillary lymph nodes (ypT0/is+ypN0)(von Minckwitz et al., 2012; Cortazar et al., 2014). Postoperative PFS was defined as the time from the start of the operation to tumor progression, death due to any reason or the last follow-up.

*Statistical analyses*

All statistical analyses were performed using Statistical Package for Social Sciences (SPSS, IBM Crop., Armok, NY,version 25) software. The χ2 test was used to determine the correlation between single/dual anti-HER-2 treatment and clinicopathological classification variables. Nonparametric tests were used to evaluate the pCR, 1-year PFS rate, 6-month PFS rate and 95% confidence interval. Nonparametric tests were used to evaluate the pCR rate, disease progression rate and 95% confidence interval. Pearson’s *χ*^2^ test and Fisher’s test were used to compare pCR, 1-year PFS, and 6-month PFS in patients from different groups. Univariate and multivariate binary logistic regression analyses were performed to analyze the association between pCR and clinicopathological variables. We reported odds ratios (ORs) and 95% confidence intervals (CIs), and P<0.05 was considered significant. The receiver operating characteristic (ROC) curve was used to determine the best clinical cut-off values of the PNI and SIRI for judging the postoperative survival status of patients, who were grouped accordingly. The Kaplan-Meier method and log-rank test were used to evaluate postoperative PFS. Univariate and multivariate Cox regression model analyses were used to determine the potential factors affecting postoperative PFS. We reported a hazard ratio (Mohri et al., 2016) and a 95% confidence interval (95% confidence interval), with significance defined as a P value <0.05. 

## Results

*Patient clinical and treatment characteristics*

Forty-seven patients with HER-2-positive breast cancer receiving neoadjuvant anti-HER-2 therapy and chemotherapy were included. Table 1 summarizes the characteristics of the included patients. The median age of the included patients was 52 years, and the median follow-up time was 10 months (calculated from the time of surgery). A total of 10 patients receiving neoadjuvant dual anti-HER-2 therapy and chemotherapy and 37 patients receiving neoadjuvant single anti-HER-2 therapy and chemotherapy were included. All patients underwent surgery and axillary lymph node dissection. All patients received anti-HER-2 therapy after surgery. Whether the patients underwent radiotherapy and/or endocrine therapy and/or chemotherapy was determined by the doctors. Notably, two of the included patients relapsed. One relapsed 3 years after surgery, and one relapsed 12 years after surgery. Both patients were in disease-free survival after the operation.

*Pathologic complete response rates*

Among the 47 patients, 13 patients achieved pCR (27.7%, 95%CI:15.6%-42.6%). The pCR rates of patients receiving neoadjuvant single and dual anti-HER-2 treatment were 24.3%, 95%CI:11.8%-41.2%) and 40.0% (95% CI: 12.2%-73.8%) (p=0.559). Univariate analysis suggested that compared with patients receiving neoadjuvant single anti-HER-2 therapy, patients receiving neoadjuvant dual anti-HER-2 therapy tended to achieve a higher pCR rate (OR=2.074, p=0.331) (Table 2). Multivariate analysis suggested that patients whose SIRI decreased more (>0.54) during NAT (p=0.027) and patients with a lower T stage (p=0.016) were more likely to achieve pCR (Table 2).

*Progression-free survival*

**Figure 1:**
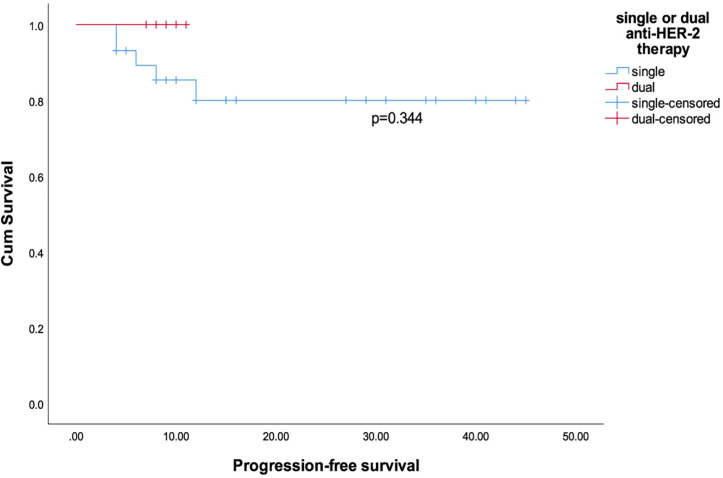
Kaplan-Meier Curves for Progression-Free Survival among Patients Receiving Neoadjuvant Single Anti-HER-2 Therapy and Chemotherapy and Patients Receiving Neoadjuvant Single anti-HER-2 Therapy and Chemotherapy

**Figure 2:**
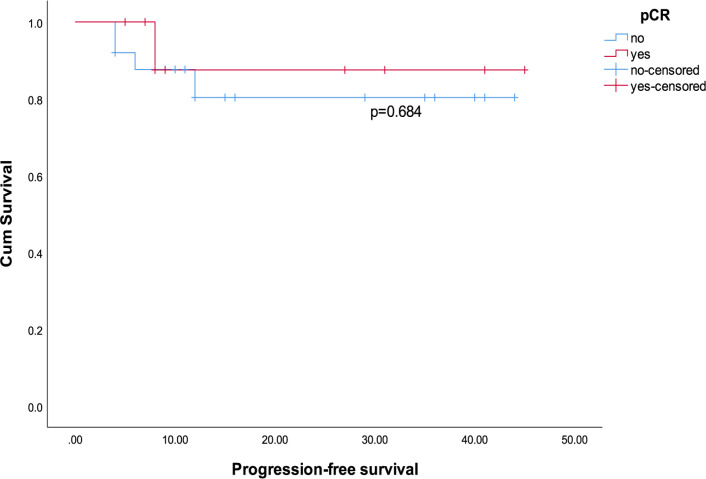
Kaplan-Meier Curves for Progression-Free Survival among Patients who Achieved pCR and Patients who did not Achieve pCR

We observed that the shortest progression time among all patients was 4 months, so we included patients with a follow-up period of ≥ 4 months. We excluded 1 patient with incomplete survival data and 1 patient with primary lung cancer. A total of 35 patients were analyzed for PFS. The median follow-up time was 12 months, and the median PFS was 10 months. Among them, 20 patients were followed for more than 1 year (all were treated with neoadjuvant single anti-HER-2 therapy and chemotherapy), and the 1-year PFS rate was 75% (95% CI: 50.9%-91.3%). All patients had a higher chance of recurrence within 6 months than after 6 months (p=0.026). The 6-month PFS rates for all patients, patients receiving neoadjuvant dual anti-HER-2 therapy and chemotherapy, and patients receiving neoadjuvant single anti-HER-2 therapy and chemotherapy were 83.9% (95% CI: 66.3%-94.5%), 100% (95% CI: 54.1%-100%) and 80% (95% CI: 59.3%-93.2%) (p=0.553), respectively. Patients who received neoadjuvant dual anti-HER-2 therapy and chemotherapy tended to have longer PFS than patients who received single anti-HER-2 therapy and chemotherapy (Figure 1, p=0.344). By the end of follow-up, the average PFS of patients who achieved pCR with neoadjuvant therapy was 18.9 months (95% CI: 7.79-30.01), while the average PFS of patients who did not achieve pCR was 16.16 months (95% CI: 10.83-21.49) (Figure 2, p =0.684). On univariate Cox regression, treatment with neoadjuvant dual anti-HER-2 and chemotherapy compared with single anti-HER-2 and chemotherapy tended to reduce the risk of disease progression (HR=0.037, p=0.556). In addition, patients who achieved pCR (HR=0.638, p=0.688) also tended to obtain longer PFS (Table 3). On the multivariate Cox regression, patients aged ≤60 years (p=0.037), with lower Ki-67 (p=0.050), and with greater SIRI reduction during NAT (>1.905) (p=0.026) had better PFS (Table 3). However, due to the large difference in the numbers of patients in the SIRI reduction high or low groups, the results may be biased.

**Table 1:** Patient, Disease and Treatment Characteristics

Variables		Total		Single anti-HER-2 therapy		Dual anti-HER-2 therapy		p
		n	(%)	n	(%)	n	(%)	
		47	100	37	78.7	10	21.3	
Age	≤60	40	85.1	31	77.5	9	22.5	1
	>60	7	14.9	6	85.7	1	14.3	
T stage	1	8	17.0	5	62.5	3	37.5	0.465
	2	23	48.9	18	78.3	5	21.7	
	3	12	25.5	11	91.7	1	8.3	
	4	1	2.1	1	100	0	0.0	
N stage	0	7	14.9	6	85.7	1	14.3	1
	1	20	42.6	16	80.0	4	20.0	
	2	4	8.5	4	100.0	0	0.0	
	3	11	23.4	9	75.0	2	25.0	
ER†	negative	30	63	25	83.3	5	16.7	0.512
	positive	17	36.2	12	70.6	5	29.4	
PR†	negative	34	72.3	30	88.2	4	11.8	0.029
	positive	13	27.7	7	53.8	6	46.2	
ER and PR†	negative	28	59.6	25	89.3	3	10.7	0.074
ER or PR†	positive	19	40.4	12	63.2	7	36.8	
HER-2†	2+	9	19.1	5	55.6	4	44.4	0.151
	3+	38	80.9	32	84.2	6	15.8	
Ki67†	<30	21	44.7	15	71.4	6	28.6	0.459
	≥30	26	55.3	22	84.6	4	15.4	
pre-NAT PNI	≤54	37	78.7	28	75.7	9	24.3	0.585
	>54	10	21.3	9	90.0	1	10.0	
	≤53.3‡	20	57.1	16	80.0	4	20.0	0.68
	>53.3‡	15	42.9	13	86.7	2	13.3	
post-NAT PNI	≤54	40	85.1	31	77.5	9	22.5	0.558
	>54	3	6.4	2	66.7	1	33.2	
	≤40.3‡	2	5.7	1	50.0	1	50.0	0.335
	>40.3‡	31	88.6	26	83.9	5	16.1	
pre-NAT PNI minus post-NAT PNI	≤9	39	83	31	79.5	8	20.5	0.226
	>9	4	8.5	2	50.0	2	50.0	
	≤4.875‡	16	45.7	13	81.3	3	18.8	1
	>4.875‡	17	48.6	14	82.7	3	17.6	
pre-NAT SIRI	≤0.785	23	48.9	16	69.6	7	30.4	0.252
	>0.785	24	51.1	21	87.5	3	12.5	
	≤0.465‡	9	25.7	6	66.7	3	33.3	0.162
	>0.465‡	26	74.3	23	88.5	3	11.5	
post-NAT SIRI	≤0.485	13	27.7	7	53.8	6	46.2	0.039
	>0.485	32	68.1	28	87.5	4	12.5	
	≤0.79‡	14	40.0	9	64.3	5	35.7	0.061
	>0.79‡	20	57.1	19	95.0	1	5.0	
pre-NAT SIRI minus post-NAT SIRI	≤0.54	38	80.9	31	81.6	7	18.4	0.35
	>0.54	7	14.9	4	57.1	3	42.9	
	≤1.905‡	32	91.4	26	81.3	6	18.8	1
	>1.905‡	1	2.9	1	100.0	0	0.0	

ER, estrogen receptor; n, number; NAT, neoadjuvant treatment; PNI, prognostic nutritional index; PR, progesterone receptor; SIRI, systemic inflammation response index, %: percentage; †, before neoadjuvant treatment; ‡, in patients included in the PFS analysis (n=35).

**Table 2:** Pathologic Complete Response

Variables		Univariate analysis			Multivariate analysis		
		p value	OR	(95% CI)	p value	OR	(95% CI)
age	≤60	ref					
	>60	0.338	2.25	(0.428-11.824)	ref		
T stage		0.015	0.211	(0.061-0.737)	0.016	0.016	(0.001-0.459)
N stage		0.612	1.189	(0.609-2.322)			
ER*	negative	ref					
	positive	0.634	0.718	(0.183-2.814)			
PR*	negative	ref					
	positive	0.768	1.235	(0.304-5.020)			
ER and PR*	negative	ref					
ER or PR*	positive	0.865	0.893	(0.241-3.308)			
HER-2*	2+	ref					
	3+	0.673	0.714	(0.150-3.408)			
Ki-67*		0.718	0.991	(0.945-1.040)			
Anti-HER-2 therapy	single	ref			ref		
	dual	0.331	2.074	(0.476-9.032)	0.98	1.055	(0.017-64.358)
pre-NAT PNI	≤54	ref			ref		
	>54	0.085	3.625	(0.837-15.703)	0.081	19.728	(0.693-561.605)
post-NAT PNI	≤54	ref					
	>54	0.828	1.318	(0.108-16.039)			
pre-NAT PNI minus post-NAT PNI	≤9	ref			ref		
	>9	0.058	10	(0.923-108.331)	0.576	0.253	(0.002-31.071)
pre-NAT SIRI	≤0.785	ref			ref		
	>0.785	0.131	2.85	(0.733-11.087)	0.469	0.308	(0.013-7.457)
post-NAT SIRI	≤0.485	ref					
	>0.485	0.585	1.515	(0.341-6.730)			
pre-NAT SIRI minus post-NAT SIRI	≤0.54	ref			ref		
	>0.54	0.016	9.375	(1.525-57.621)	0.027	1445.425	(2.322-899671.5)

CI, confidence interval; ER, estrogen receptor; NAT, neoadjuvant treatment; OR, odds ratio; PNI, prognostic nutritional index; PR, progesterone receptor; ref, reference; SIRI, systemic inflammation response index; *, before neoadjuvant treatment.

**Table 3:** Progression-Free Survival

Variable		Univariate Cox Regression			Multivariate Cox Regression		
		p value	HR	(95% CI)	p value	HR	(95% CI)
age	≤60	ref			ref		
	>60	0.176	3.45	(0.574-20.745)	0.037	134.484	(1.348-13418.243)
T stage		0.158	2.213	(0.734-6.617)	0.108	9.417	(0.611-145.129)
N stage		0.917	0.954	(0.393-2.314)			
ER*	Negative	ref					
	Positive	0.338	0.342	(0.038-3.070)			
PR*	Negative	ref					
	Positive	0.814	0.766	(0.084-6.991)			
ER and PR*	Negative	ref					
ER or PR*	Positive	0.292	0.307	(0.034-2.766)			
HER-2*	2+	ref					
	3+	0.761	0.712	(0.079-6.392)			
Ki-67*		0.109	1.043	(0.991-1.099)	0.049	1.133	(1.001-1.283)
Anti-HER-2 therapy	Single	ref			ref		
	Dual	0.556	0.037	(0.0-2209.285)	0.987	0.001	(0.0-0.0)
pre-NAT PNI	≤53.3	ref					
	>53.3	0.468	1.941	(0.323-11.667)			
post-NAT PNI	≤40.3	ref					
	>40.3	0.727	22.299	(0.0-84973360.8)			
pre-NAT PNI minus post-NAT PNI	≤4.875	ref					
	>4.875	0.303	68.79	(0.022-215861.8)			
pre-NAT SIRI	≤0.465	ref					
	>0.465	0.453	30.413	(0.004-226078.6)			
post-NAT SIRI	≤0.79	ref					
	>0.79	0.334	43.9	(0.020-94355.0)			
pre-NAT SIRI minus post-NAT SIRI	≤1.905	ref			ref		
	>1.905	0.014	32	(2.002-511.602)	0.026	198.665	(1.870-21110.073)
pCR	No	ref			ref		
	Yes	0.688	0.638	(0.071-5.715)	0.931	1.128	(0.074-17.110)

CI, confidence interval; ER, estrogen receptor; HR, hazard ratio; NAT, neoadjuvant treatment; pCR, pathologic complete response; PNI, prognostic nutritional index; PR, progesterone receptor; ref, reference; SIRI, systemic inflammation response index; *, before neoadjuvant treatment.

## Discussion

The purpose of this retrospective analysis was to evaluate the efficacy of neoadjuvant anti-HER-2 therapy combined with chemotherapy in unselected Chinese patients with HER-2-positive breast cancer. We found that patients with a lower T stage were more likely to achieve pCR. Patients with advanced T stage of tumors, tended to have worse PFS. This was consistent with the results of other previous studies (Fayanju et al., 2018; Prat et al., 2020). In addition, patients aged ≤60 years or with lower Ki-67 had better PFS. Other studies also showed that younger patients and patients with lower KI-67 were more likely to get longer PFS (Klauschen et al., 2015; Ignatiadis et al., 2019; Yu et al., 2019; Kanjanapan et al., 2020). The outcomes also showed that patients treated with neoadjuvant chemotherapy combined with dual anti-HER-2 therapy tended to obtain a higher pCR rate and better PFS than those treated with chemotherapy and single anti-HER-2 therapy. Patients who achieved pCR also tended to have a lower risk of disease progression. This was consistent with the results of the NeoSphere study. The NeoSphere study found that compared with patients receiving trastuzumab plus docetaxel, patients who were given pertuzumab and trastuzumab plus docetaxel had a significantly higher pCR rate with no significant difference in toxicity (Gianni et al., 2012). The 5-year follow-up results of this trial showed that patients with pCR had a longer PFS rate than those without pCR (5-year PFS rate was 85% for those with pCR and 76% for those without pCR, HR=0.54, 95% CI: 0.29 1.00) (Gianni et al., 2016). Similarly, the results of the Asian population subgroup analysis in the PEONY phase 3 randomized clinical trial also showed that compared with neoadjuvant trastuzumab and docetaxel, neoadjuvant pertuzumab and trastuzumab plus docetaxel therapy significantly increased the pCR rate of early or locally advanced HER-2-positive Asian breast cancer patients (Shao et al., 2020). Notably, in our study results, the p values for the pCR rate and PFS for single/dual anti-HER-2 therapy and the p value of PFS for patients with pCR were both greater than 0.05, indicating that there was no significant difference, which may be related to factors such as the small sample size of the included population, the insufficient follow-up time, and the timing of surgery. The National Surgical Adjuvant Breast and Bowel Project Protocols B-27 and its updated results indicated that patients with neoadjuvant chemotherapy who completed the total number of courses of adjuvant chemotherapy before surgery had a higher pCR rate than those who divided chemotherapy courses into preoperative chemotherapy and postoperative chemotherapy, while patients who achieved pCR had better disease-free survival (DFS) and overall survival (OS) (Bear et al., 2003; Rastogi et al., 2008). However, due to the patients’ will and the limitations of the patients’ economic conditions and the medical environment in China, for some chemotherapy regimens that require the completion of 8 cycles before surgery, there were cases in China in which surgery was performed in advance after the completion of 4 cycles, and the remaining 4 cycles of chemotherapy were completed after surgery. Not all patients who did not achieve pCR underwent further chemotherapy after surgery. This could affect the results of the study. We will continue to follow the prognosis of patients to further determine the optimal timing of surgery. Larger studies are needed to further evaluate the efficacy of neoadjuvant anti-HER-2 therapy combined with chemotherapy in Chinese patients with HER-2-positive breast cancer and the selection of surgical timing for patients receiving neoadjuvant chemotherapy combined with anti-HER-2 treatment.

Numerous studies have shown that tumors are associated with systemic inflammation (Romero-Cordoba et al., 2019; Tuomisto et al., 2019; Fest et al., 2020). As a simple and easily available prognostic indicator, the SIRI shows certain prognostic value in many tumors (including breast cancer) (Qi et al., 2016; Sun et al., 2020). Our study suggested that patients with greater SIRI reduction (>0.54) during neoadjuvant therapy had a higher pCR rate (p= 0.027). Similarly, some studies showed that in esophageal cancer and cervical cancer, patients whose SIRI decreased more after surgery may have a better prognosis (Geng et al., 2018; Chao et al., 2020). Hua et al. found that a lower SIRI (<0.54) was a good prognostic factor for postmenopausal breast cancer patients (p=0.008) (Hua et al., 2020). Chen et al. found that a lower SIRI (<0.85) in breast cancer patients before NAC indicated better DFS and OS (p=0.011, p=0.017)(Chen et al., 2020). Wang et al. found that the OS rate of breast cancer patients with an SIRI<0.65 before or after surgery was significantly higher than that of breast cancer patients with an SIRI>0.65 (p<0.001) (Wang et al., 2020b). Although our study did not show that the more decrease in SIRI during neoadjuvant therapy was associated with longer PFS. This may because patients may have drug-induced leukopenia while receiving neoadjuvant therapy, and the overuse of drugs that raises leukocytes could affect SIRI. As the treatment becomes more and more standardized, we will continue to follow up more patients to obtain more accurate results in the future. The possible reason that the SIRI affects the prognosis of patients is that neutrophils can promote tumor proliferation and metastasis and can release some cytokines and chemokines to suppress the immune system (Laviron et al., 2019). Monocytes, especially TAMs, have an impact on the tumor microenvironment by promoting tumor progression and metastasis (Gregory and Houghton, 2011). Lymphocytes are an important part of antitumor immunity and immune surveillance and inhibit tumor cell proliferation and migration by inducing cytotoxic cell death (Mantovani et al., 2008). Therefore, a lower SIRI might predict better outcomes for breast cancer patients. More clinical data are still needed to verify this view.

Many studies found that a high PNI was an independent positive prognostic factor in many tumors (such as cancer and lung cancer) (Tokunaga et al., 2015; Nakatani et al., 2017; Jin et al., 2018; Wang et al., 2020a). In breast cancer, Mohri et al. also found that patients with high preoperative PNI status had a better 5-year OS rate (p = 0.013) (Mohri et al., 2016). Oba et al., (2020) found that excessive PNI reduction during NAC in breast cancer patients (>5.26) was related to poor prognosis in breast cancer, suggesting that breast cancer patients who maintain a better nutritional status during NAC may obtain better effects. The univariate analysis in our results suggested that patients with a higher PNI before neoadjuvant therapy or surgery tended to have a higher pCR rate and longer PFS, but these results were not statistically significant. This may be due to the limited sample size included. More studies are needed to evaluate the significance of the PNI in the treatment of breast cancer patients with neoadjuvant chemotherapy combined with anti-HER-2 drugs.

Accurately predicting the prognosis of breast cancer patients is of great significance. It can help clinicians develop more appropriate treatment plans. Our study is a preliminary evaluation of the efficacy of neoadjuvant anti-HER-2 therapy and chemotherapy in Chinese patients with HER-2-positive breast cancer, and more studies are needed to comprehensively evaluate the prognosis of patients.

*Limitations*

1) Due to the availability of drugs, patient economic conditions, and medical insurance coverage, breast cancer patients in our center have only gradually begun to use anti-HER-2 drugs in recent years, especially dual-anti-HER-2 therapy. The time that these drugs were used for neoadjuvant therapy was even shorter. As a single-center retrospective study, the sample size was small. The follow-up time was relatively short. These may lead to biased results. The small sample size may make certain factors that enable patients to obtain higher pCR rates and longer PFS only show corresponding trends in this study, and may also cause the interference effects of confounding factors to be amplified. In addition, many patients had no tumor progression at the time of follow-up, so the exact time of PFS was not available. By the end of follow-up, only 1 patient died, so we could not assess the OS. We would expand the sample size in the future and continue to follow up to further verify our results. More prospective studies are needed to evaluate the efficacy of neoadjuvant anti-HER-2 therapy and chemotherapy in Chinese patients with HER-2-positive breast cancer.

2) The population we included may have a combination of different neoadjuvant chemotherapy regimens, and some Chinese breast cancer patients started surgery early after completing only 4 cycles of treatment, which may influence the results. And due to poor recovery of hand function after surgery, some patients may not be able to receive radiotherapy on time. This may also affect the patient’s OS. Further studies are needed to evaluate the effects of the neoadjuvant anti-HER-2 combined chemotherapy regimen and surgical timing on the chemotherapy response and prognosis of breast cancer patients in China.

3) As a retrospective study, it has some inherent limitations. The outcomes were hypothesis generating and showed that patients with greater SIRI reduction (>0.54) during neoadjuvant therapy and patients with a lower T stage had higher pCR rates. Patients who were ≤60 years old, had lower Ki-67 had longer PFS. Patients receiving neoadjuvant dual anti-HER-2 therapy and chemotherapy tended to have a higher pCR rate and better PFS. Patients with pCR also tended to obtain better PFS.

In conclusion, the greater reduction in SIRI during NAT indicated the greater possibility of achieve pCR. Patients receiving neoadjuvant dual anti-HER-2 therapy and chemotherapy had a tendency to reach pCR and had a longer PFS. It is very important to accurately predict the patient’s prognosis, which helps make clinical decisions. We will continue to follow up more patients to verify our hypothesis.

## Author Contribution Statement

(I) Conception and design: All authors; (II) Administrative support: None; (III) Provision of study materials or patients: QH, QR; (IV) Collection and assembly of data: QH, JL; (V) Data analysis and interpretation: QH; (Perez et al.) Manuscript writing: All authors; (VII) Final approval of manuscript: All authors. 
